# Stress Induced Hormone and Neuromodulator Changes in Menopausal Depressive Rats

**DOI:** 10.3389/fpsyt.2018.00253

**Published:** 2018-06-13

**Authors:** Simeng Gu, Liyuan Jing, Yang Li, Jason H. Huang, Fushun Wang

**Affiliations:** ^1^School of Psychology, Jiangsu University Medical Center, Zhenjiang, China; ^2^School of Psychology, Institute of Emotional Studies, Nanjing University of Chinese Medicine, Nanjing, China; ^3^Department of Neurosurgery, Baylor Scott & White Health, Temple, TX, United States; ^4^Department of Surgery, Texas A&M University, Temple, TX, United States

**Keywords:** ACTH, cortisone, menopausal, LH, FSH, monoamine, depression

## Abstract

**Objective:** Previously, we showed that neuromodulators are important factors involved in depression, here we aim to further investigate the interactions between neuromodulators and sex hormone involved in menopause related depression in rats.

**Methods:** Menopausal depression was made with bilateral ovariectomies in female SD rats followed by chronic mild unpredictable stress treatment for 21 days. Thirty six rats were randomly divided into four groups: sham surgery group, sham/stress group, surgery group, surgery/stress group. Then open-field locomotor scores and sucrose intake were employed to observe behavior changes. The levels of norepinephrine (NE), dopamine (DA), serotonin (5-HT) in the cerebral spinal fluid and serum adrenocorticotropic hormone (ACTH), cortisone were determined with High-performance liquid chromatography (HPLC). Serum estradiol (E2), follicle-stimulating hormone (FSH), and luteinizing hormone (LH) were measured with radioimmunoassay.

**Results:** The open-field locomotor scores and sucrose intake were significantly decreased after the surgery and stress treatment (*p* < 0.01). The Serum E_2_ level decreased significantly after the surgery (*p* < 0.01), but serum LH, FSH levels increased significantly in the surgery group than the sham surgery group (*p* < 0.01). The cortisone levels increased significantly in sham/stress group than that in the sham surgery group during the first 2 weeks at stressful treatment, but decrease afterwards. The monoamine levels in the surgery/stress group were much lower than those in the sham surgery group (*p* < 0.01). The correlation analysis found that LH and FSH are related more to the neurotransmitter release than E_2_.

**Conclusion:** Ovary removal rats showed depression-like behaviors, with LH and FSH increase and monoamine decrease, and the levels of these monoamines in the stress treated groups changed only after the stressful treatment. The LH, FSH hormone increasing might be the reason for the lower monoamine release, which in turn might be the reason for depressed syndromes in the menopause. The cortisone and ACTH in the serum in the surgery/stress group were much higher than that in the sham surgery group.

## Introduction

Major depressive disorder is a leading cause of disability worldwide, and affects more than 17% of the populations, making it one of the most prevalent health-related causes of human suffering (1, 2). However, the mechanisms of depression are far from clear (3), the most widely accepted theory about the mechanism of depression points to the monoamine neurotransmitters, including norepinephrine (NE), serotonin (5-HT), and dopamine (DA) (4–6). The 5-HT and NE reuptake inhibitors and selective 5-HT reuptake inhibitors are considered to be the first choice of treatment for depression (2, 7–9). Even though compelling evidence from genetic studies and pharmacological studies points to dysfunction of the central monoamine network in depression (10), many other hormones or neuromodulators might be involved too, such as hypothalamus-pituitary-axis (HPA) hormones (1) and sex hormones (11).

Depression occurrence in female patients are twice as many as male patients (12), which suggested that depression is related to sex hormone (13, 14). In addition, menopause depression is a well-known symptom for menopausal period (15, 16), which is due to the ovarian dysfunction 1 year after menopause (17). The major symptom of menopause depression is similar to that of major depression (18, 19). However, after decades of accumulated research, the existence of a menopause-associated depression is still controversial. The confusion is attributed to insufficient knowledge on the women's “vulnerability” to depression in the midlife years (19). Here we screened the chronic changes of hormones and neurotransmitters after artificial removal ovaries in adult rats, trying to see how the hormone changes and their interaction with monoamine neurotransmitters. This study would help us clarify the relationship between sex hormones and neurotransmitters.

## Materials and methods

### Animals

Thirty six female Sprague-Dawley (SD) rats with body weight averaged (470 ± 20) g, at the age about 1 years old, were purchased from Academy of Military Medical Sciences (Beijing, China). They were randomly divided into 4 groups: sham surgery group (faked surgery with no stress), sham/stress group (faked surgery with stress), surgery group (surgery with no stress treatment), surgery/stress group (surgery with stress treatment), with 9 rats in each group. The animals were treated as shown before: 9 rats in the sham surgery group were treated with sham surgery (only open the abdomen), 9 rats in the sham/stress group were treated with sham surgery and stress, 9 rats in the surgery group were only treated with surgery to remove the ovaries but no stress, 9 rats in the surgery/stress group were treated with surgery and stress. After 1 week's recovery from the surgery, the animals were treated with chronic unpredictable mild stress for 3 weeks. All the procedures were approved by the Institution of Animal Care and Use Committee of Nanjing University of Chinese Medicine.

### Instrument

US thermo microplate reader, which was used to read the radioimmunoassay; KH30R desktop high-speed refrigerated centrifuge, low temperature ultracentrifuge, which are used for taking up serum, are provided by the central laboratory. 3200 ATRAP high-performance ligquid chromatography (HPLC) tandem mass spectrometer (ABI, USA), equipped with atomospheric pressure chemical ionization sources (LC-APCI-MS/MS).

### Animal surgery/control

#### Ovary removal

Rat in the two surgery groups were given “ovariectomy,” and the rats in the sham surgery groups were just given surgery to open the belly. Surgical procedure: the surgery was performed in a super-clean bench, and the rats were given 100 mg.kg^−1^ ketamine to induce anesthesia and fixed on a hard plate with supine position. Seventy-five percentage alcohol was used to clean the skin of surgery place. A marker was made in the rat 1 cm below the ribs, and 2 cm outside the spine and the soft tissue was sectioned separately. The peripheral blood vessels were ligated, and both sides of ovaries were removed, and finally the uterus was removed, and the skin was sutured, and the rats were put in one cage to be fed separately. It usually took 15 min for the surgery, and all the rats survived after the surgery. The method of determining successful ovariectomy is the vaginal epithelium keratosis test: 5 days continuous monitoring the rat vagina did not find the estrous cycle.

#### Chronic unpredictable mild stress

All rats were housed in a single cage after the surgery, and after 7 days' recovery from surgery, they were treated with random chronic unavoidable stresses for 21 days. Stress treatment lasted for the 2 h, which includes 36 V AC electric shock foot (every l min stimulated 1 times; each time lasted 10 s, a total of 30 times), 4°C cold water swimming 5 min, 45°C heat stress 5 min, 15 min shaking (1 times/s), rat cage tilted 45° for 12 h, clip tail (1 min), wet bedding 10 h, empty bottle for 1 h, each stimulus applied for 2 times. Rats in stress group were treated with stress continuously for 21 days after the surgery.

### Behavioral observation indicators

#### Open-field test

Every 7th day after the surgery, the animals were tested with “open-field test,” which was done in an open box as reported before (20), with the height to be 40 cm, and both the length and width to be 80 cm. The bottom surface was marked with white lines which divided into 25 large areas. The horizontal activity of rats was measured by the number of bottom blocks, 1 grid is 1 point, and the vertical activity points were measured as the number of upright, from the rat's front feet left the bottom to lay down their feet. Testing was performed for 5 min in a well-illuminated transparent acrylic cage, and the rats were gently placed in the center and left to explore the area for 5 min. The digitized image of the path taken by each animal was tracked by a camera, and the total locomotor activity was analyzed using ANY-maze software. The testing apparatus was cleaned with 70% ethanol and then dried between each test.

#### Sucrose intake test

Sucrose intake test was carried out on the 7th day after surgery. Each rat was given 133 mL of 1% sucrose solution after 24 h fasting and the amount of sucrose solution consumed by rats was calculated.

### Hormones and neurotransmitters measurement

Estradiol (E2), luteinizing hormone (LH) and follicle-stimulating hormone (FSH) were measured every 7th day from the blood serum from the caudal vein. At about 10 O'clock every morning on the 7th day, 500 μl blood was driven from the caudal vein from each animal with a 1 ml syringe, which was washed with 100 μl 0.9% NaCl and 12500 U heparin inside. The supernatant was ready to be used for the radioimmunoassay after the blood was centrifuged. The content of E2 (estradiol), LH (luteinizing hormone) and FSH (follicle-stimulating hormone) were determined by radioimmunoassay. RIA kit used was bought from the United States Depp Company. Specific operation was carried out according to the instructions. The ACTH and cortisone in the serum were measured with a 3200 ATRAP high-performance liquid chromatography (HPLC) tandem mass spectrometer (ABI, USA), equipped with atmospheric pressure chemical ionization sources (LC-APCI-MS/MS). The neurotransmitter measurement in the cerebral spinal fluid was also done with HPLC. The cerebral spinal fluid (CSF) was dilated with a pump to inject artificial CSF to the brain and the CSF pumped out was used in a HPLC to measure NE, DA and serotonin (21). The monoamine levels were assessed by comparing the reference standard with respective peak area and elution time of the samples using a calibration curve for each monoamine neurotransmitter. After the chronic stress and the behavior test, the animals were decapitated under anesthesia with isoflurane (2%).

### Statistical processing method

SPSS20.0 software was used for statistical analysis, and the data were used in the form of $\bar{\text{x}}$ ± s. Analysis of variance (ANOVA) and repeated measured ANVOA were used to compare the difference between groups.

## Results

### Body weight

The body weight was measured before the surgery and also on the 7th day after surgery. Table 1 shows that the body weights of the animals in each group decreased than those in the sham surgery group, and the body weights of the rats in the sham/stress and surgery/stress groups were significantly lower than those in the sham surgery group (*p* < 0.01, One-Way ANOVA, Table 1), but there was no significant difference between the sham surgery group and surgery group (*p* > 0.05, One-Way ANOVA,).

**Table 1:** Stress on body weight of rats with menopausal depression (*x* ± *s, g*).

**Group**	**Sham surgery**	**Sham/stress**	**Surgery**	**Surgery/stress**
Before castration	474.53 ± 11.49	475.29 ± 8.15	479.15 ± 9.94	473.49 ± 8.75
After castration	471.32 ± 9.34	456.31 ± 9.43^*^	462.69 ± 8.28	447.73 ± 7.15^*^

* *p < 0.01, One-way ANOVA*.

### Behavioral assessment

**Figure 1:**
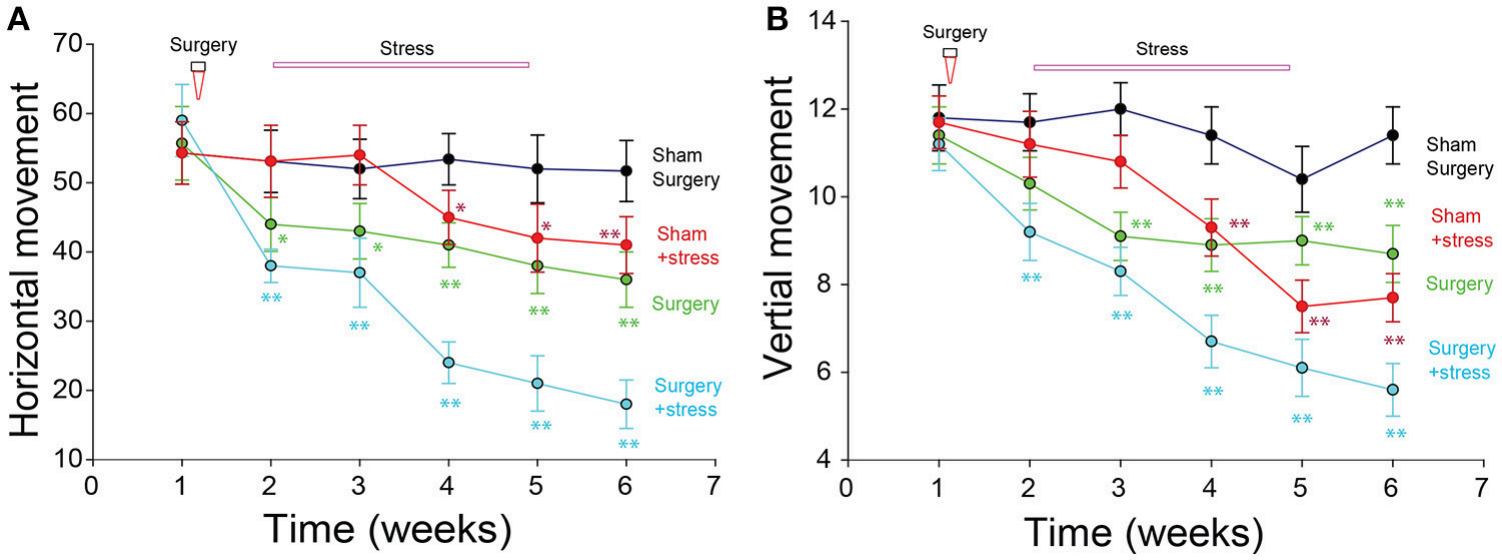
Open box movement changes after surgery and stress treatment. **(A)** Both the stress and surgery decreased the horizontal movement. **(B)** Both the stress and surgery decreased the vertical movement (^*^p < 0.01, ^**^p < 0.001, repeat measure one-way ANOVA comparisons between the four groups on the 7th days).

The results showed that there was no significant difference before the surgery or stressful treatment in the behavior tests among the surgery groups (One-way ANOVA, *p* > 0.05, Figure 1). But the scores of vertical movement and horizontal movement in the surgery groups decreased gradually than those in the sham surgery group (repeated measures one-way ANOVA, Normality test passed *p* = 0.031; and the differences among the four different treated groups were significantly higher, and they were dependent on the time). We next compared the four different treated groups, and found that the movements in surgery and stress treated groups were different from that of the sham group, (^*^p < 0.01, ^**^p < 0.001, One-way ANOVA comparisons between the four groups on the same 7th days), but there was no significant difference between the sham + stress group and surgery only group.

### Sucrose intake

**Figure 2:**
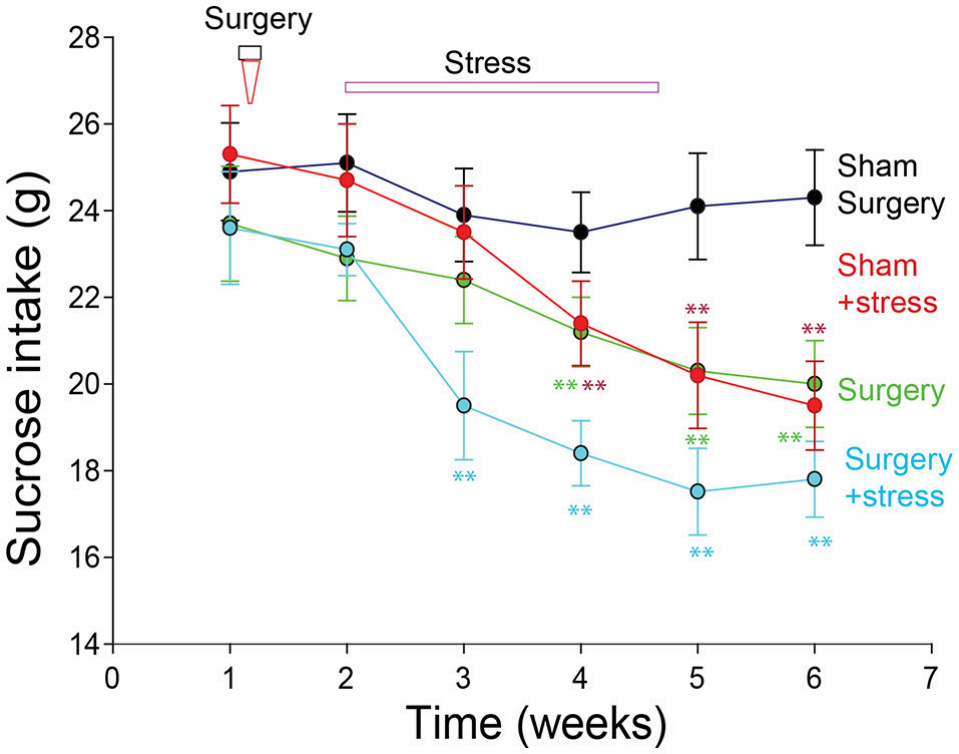
Sucrose intake in rats with menopausal depression (^*^p < 0.01, ^**^p < 0.001, repeated measure one-way ANOVA comparisons between the four groups on the 7th days).

The rats in the castration group consumed less sucrose than those in the sham surgery only group (*p* < 0.001, One-way ANOVA comparisons between the four groups on the 7th days; Figure 2). The sucrose consumption in the stress + surgery group was even lower than that in the stress and sham surgery group (*p* < 0.001, One-way ANOVA). Repeated measure one-way ANOVA was done to find that Normality test passed *p* = 0.086; and there is significant difference among the four different treated groups, especially the surgery + stress group compared with the sham surgery with no stress treatment group.

### Monoamine neurotransmitters and hormones

**Figure 3:**
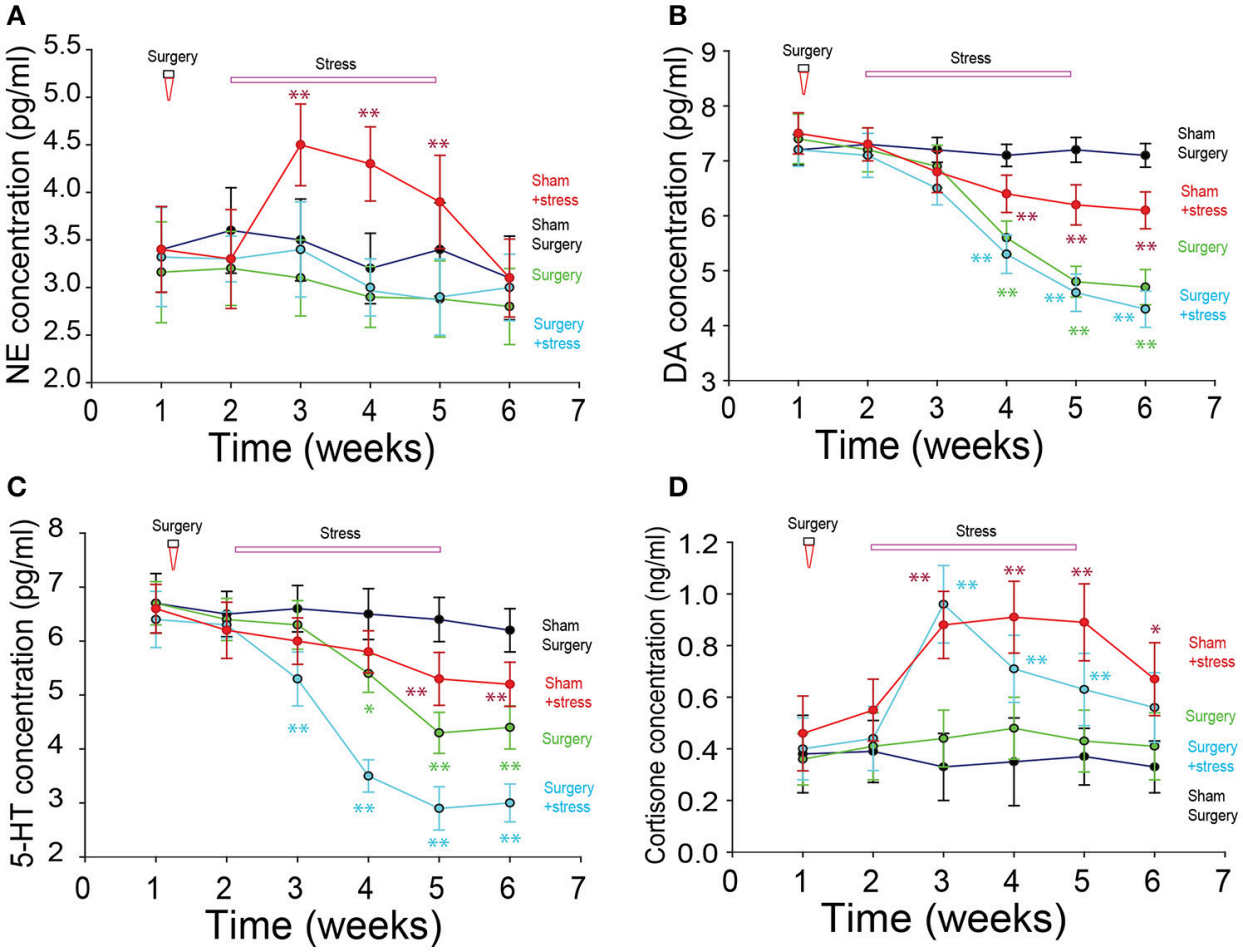
Neuromodulator changes in rats with menopausal depression. **(A)** Norepinephrine (NE) changes did not change much among the groups (*P* > 0.05, One-way ANOVA). **(B)** Dopamine (DA) decreased significantly after the stress and surgery treatment. **(C)** 5-HT decreased significantly after the stress and surgery treatment. **(D)** Cortisone increased significantly in the stress treatment groups (^*^p < 0.01, ^**^p < 0.001, repeat measure one-way ANOVA comparisons between the four groups on the 7th days).

The levels of neurotransmitters norepinephrine (NE), dopamine (DA) and serotonin (5-HT) in the CSF and cortisone in the serum were tested with high performance liquid chromatography (HPLC). Repeated measure one-way ANOVA was done in NE, DA, 5-HT and cortisone group respectively; and there were significant differences among the four different treated groups, especially the surgery + stress group compared with the sham surgery with no stress treatment group). We next compared the four different treated groups, and found that the concentrations of NE, DA, 5-HT and cortisone in surgery and stress treated groups were different from the sham group, starting from the first week stress treatment (^*^p < 0.01, ^**^p < 0.001, One-way ANOVA comparisons between the four groups on the same 7th days). The levels of NE and cortisone in the CSF increased significantly lower in the surgery groups compared with the sham surgery group, and the concentration of DA and 5-HT increased significantly in the surgery groups starting from the first week stress treatment. The cortisone levels in the serum in the sham/stress group were much higher than that in the sham surgery group. And the levels of these monoamines in the stress treated groups changed only after the stressful treatment (^*^p < 0.01, ^**^p < 0.001, repeated measure one-way ANOVA comparisons between the four groups; Figure 3).

### Sex hormones

**Figure 4:**
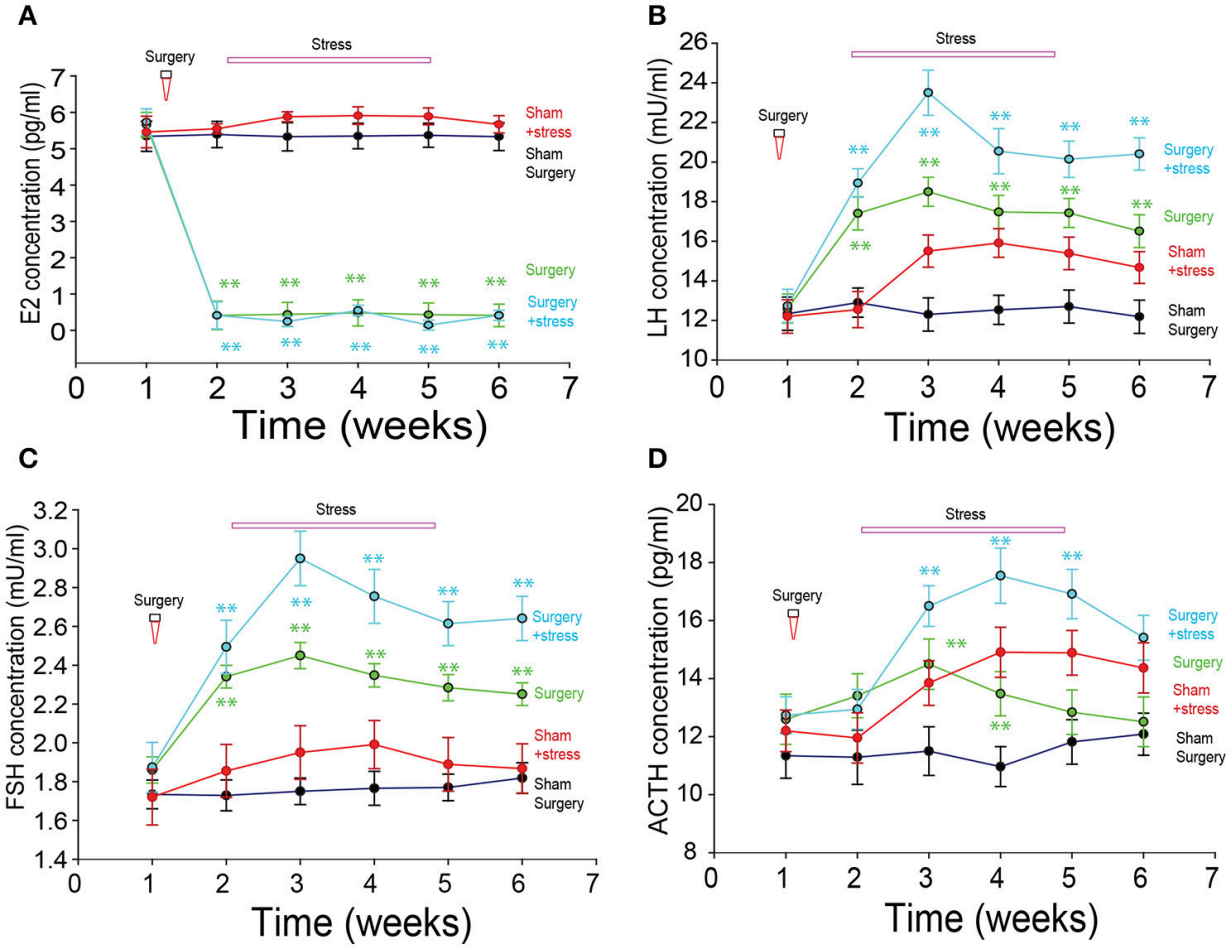
The sex hormone changes after bilateral ovariectomies. **(A)** Estradiol changes among the groups. **(B)** Luteinizing hormone (LH) changes among the groups. **(C)** Follicle-stimulating hormone (FSH) changes among the groups. **(D)** ACTH changes among the groups. (^*^p < 0.01, ^**^p < 0.001, repeated measure one-way ANOVA comparisons between the four groups on the 7th days).

The levels of estradiol in the serum of the surgery group were significantly lower than those in the sham surgery group (repeated measure one-way ANOVA, *p* < 0.01; Figure 4), but they were not different between the sham surgery and sham surgery/stress group or surgery group with surgery/stress group. The levels of serum luteinizing hormone and follicle-stimulating hormone in the two stressful groups were significantly higher than those in the sham surgery group (repeated measure one-way ANOVA, *p* < 0.01). The level of serum ACTH was significantly higher in the stressful treatment group than that in sham surgery group (repeated measure one-way ANOVA, *p* < 0.01).

### Correlation between sex hormones and neurotransmitters

The correlation between the levels of sex hormones and neurotransmitters was analyzed, and we found that there was little correlation between estradiol in the serum with the neurotransmitter, but the correlations were significantly high in the two stress treated groups (Table 2).

**Table 2:** Correlations between sex hormones with neuromodulators.

**Group**	**Estradiol**	**LH**	**FSH**
NE	0.12	0.54	0.50
DA	0.07	−0.34	−0.36
5-HT	0.14	−0.28	−0.27
Cortisone	0.16	0.65	0.68
ACTH	0.04	0.72	0.76

## Discussion

The pathological mechanism of depression is far from clear (3, 22), and is a hot topic for neuroscience research (23, 24). We have reported before that patients with depression in the brain showed monoamine neurotransmitter changes and neurological dysfunction in the cortex and the hippocampus (1, 2). However, there are many other hormones that also involved (22), such as hormones released from hypothalamus-pituitary-axis (HPA) (25, 26). In this study, we probed into the role of sex hormones in the depression, because clinical data showed that the occurrence of depression in female patients are twice as many as male patients (25), which suggested that estradiol might induce depression (27). However, it seems contradictory that depression is a well-known symptom for perimenopausal period, which suggested that lack of estradiol will induce depression (28). Here, we used ovariectomized female SD rats to stop the estradiol release, and found that these rats were much easier to get depressed at chronic mild stress. On the contrary to decreases in estradiol release, LH and FSH levels are greatly increased, possibly due the removal of feedback inhibition of estradiol (29). In addition, the correlation analysis found that LH and FSH are positively correlated with the changes of stress hormones such as cortisone and ACTH. Therefore the LH and FSH increase might be the reason for depression. As far as we know, this study might be the first to directly measure the chronic changes of the sex hormones and also neurotransmitters after ovary removal and also chronic stress.

### The stress related hormones increased together with LH and FSH

In addition to LH and FSH, the cortisone level also increased after the ovary removal, especially in the face of stressful situations. Consistent with our reports, abnormal cortisone release following stress has been reported (30). ACTH and cortisone, which are part of HPA axis (Hypothalamus-pituitary-adrenal axis) are stressful hormones (30). The positive correlation between LH and FSH together with cortisone and ACTH suggests that LH and FSH are related to stress. LH, FSH and ACTH are all released from anterior pituitary gland, which are regulated by neurons in the hypothalamus, which in turn are negatively controlled by estradiol. There is also the possibility for LH and ACTH to coordinate with each other to enhance the stressful reactions, which might be reason for the menopause depression. If so, it is easy to understand women's prone to depression. Consistent with the increase of LH and FSH after ovariectomization, who are also increased in the menopause patients (31, 32). LH release surges up during ovulation in the menstrual cycle, and LH detection is used to detect ovulation, which occurs about 24–48 h after the LH surge. LH surge up during ovulation in the menstrual cycle and also after menopause might be the reason for depression.

### The stress related norepinephrine also increased together with LH and FSH

In addition to ACTH and cortisone, NE also increased after stressful treatment and ovariectomization. It is easy to understand the increase of cortisone, which is the downward pathway for ACTH. But how does NE release was affected is not clear. There is also the possibility that HPA axis affects NE release. Maybe, LH can also directly affect the brain functions (32), many papers have focused on the effects of estradiol on the monoamine release or up-take (33). Estradiol has profound effects on brain chemistry, structure, and function and is trophic for regions such as the pre-frontal cortex and hippocampus that are critical to affect emotional regulation (34). LH was recently reported as a candidate for its role in the mental disorders (12, 35).

### The reward related DA and 5-HT neurotransmitter release are negatively correlated with LH and FSH

Contrary to increases of NE and ACTH release, DA and 5-HT releases decreased significantly. DA and 5-HT are important neurotransmitters in the brain, and low level of either DA or 5-HT has proved to be related to depression (7, 36–38). DA is the neurotransmitter for reward (39), and low level of DA function can lead to depression (40, 41). The serotonin transporter ensures the recapture of serotonin and is the pharmacological target of selective serotonin reuptake inhibitor (SSRI) antidepressants (42). Our data found that both DA and 5-HT releases are negatively correlated with LH release after ovariectomization, which further support the role of LH in depression. The reason might be that the projections from neurons in hypothalamus inhibits the DA release (43–45), or 5-HT release. Another possible way is that estradiol and LH might directly affect the DA neurons (38). Sure there might be interactions between monoamine neurons and hypothalamus (46). However, our data showed that the LH increase seems to start at the first 7th days, while the DA and 5-HT release decrease tops at the 3rd 7th days, which suggested that LH increase might be the cause for the DA and 5-HT decrease.

In all, perimenopause and postmenopause provide a natural laboratory for the study of sex hormone on depression (16). With the aging of our society, women living at least a third of their lives in the postmenopausal state, determining the long-term health effects of hormonal fluctuations has become a major women's health concern.

## Author contributions

FW and JH designed the experiments. SG, LJ, and YL performed the experiments. SG and LJ did the data analysis. FW and JH wrote the paper.

### Conflict of interest statement

The authors declare that the research was conducted in the absence of any commercial or financial relationships that could be construed as a potential conflict of interest.

## References

[1] Wang F Pan F Lee L. A Huang J. H. Stress induced neuroplasticity and mental disorders. Neural Plast. (2017) 2017:9634501. 10.1155/2017/9634501 28785487 PMC5530430

[2] Gu S Wang W Wang F Huang J. H. Neuromodulator and Emotion Biomarker for Stress Induced Mental Disorders. Neural Plast. (2016) 2016:2609128. 10.1155/2016/2609128 27051536 PMC4808661

[3] Malinow R Depression: Ketamine steps out of the darkness. Nature (2016) 533:477–8. 10.1038/nature17897 27144350 PMC5102012

[4] Moeller SJ Parvaz MA Shumay E Wu S Beebe-Wang N Konova AB. Monoamine polygenic liability in health and cocaine dependence: imaging genetics study of aversive processing and associations with depression symptomatology. Drug Alcohol Depend (2014) 140:17–24. 10.1016/j.drugalcdep.2014.04.019 24837582 PMC4053494

[5] Hamon M Blier P. Monoamine neurocircuitry in depression and strategies for new treatments. Prog Neuro-Psychopharmacol Biol Psychiatry (2013) 45:54–63. 10.1016/j.pnpbp.2013.04.009. 23602950

[6] Marshe VS Maciukiewicz M Rej S Tiwari AK Sibille E Blumberger DM. Norepinephrine transporter gene variants and remission from depression with venlafaxine treatment in older adults. Am J Psychiatry (2017) 174:468–75. 10.1176/appi.ajp.2016.16050617. 28068779

[7] Muhammed K Manohar S Ben Yehuda M Chong TT-J Tofaris G Lennox G. Reward sensitivity deficits modulated by dopamine are associated with apathy in Parkinson's disease. Brain (2016) 139:2706–21. 10.1093/brain/aww188 27452600 PMC5035817

[8] Zheng Z Gu S Lei Y Lu S Wang W Li Y. Safety needs mediate stressful events induced mental disorders. Neural Plast. (2016) 2016:8058093. 10.1155/2016/8058093 27738527 PMC5050353

[9] Zahavi AY Sabbagh MA Washburn D Mazurka R Bagby RM Strauss J. Serotonin and dopamine gene variation and theory of mind decoding accuracy in major depression: a preliminary investigation. PLoS ONE (2016) 11:e0150872. 10.1371/journal.pone.0150872 26974654 PMC4790964

[10] Wright B Alexander D Aghahoseini A York Surgical Outcomes Research Team. Does preoperative depression and/or serotonin transporter gene polymorphism predict outcome after laparoscopic cholecystectomy? BMJ Open (2016) 6:e007969. 10.1136/bmjopen-2015-007969 27601483 PMC5020877

[11] Freeman EW. Associations of depression with the transition to menopause. Menopause (2010) 17, 823–7. 10.1097/gme.0b013e3181db9f8b 20531231

[12] Blair J. A Palm R Chang J McGee H Zhu X Wang X Luteinizing hormone downregulation but not estrogen replacement improves ovariectomy-associated cognition and spine density loss independently of treatment onset timing. Horm Behav. (2016) 78:60–66. 10.1016/j.yhbeh.2015.10.013 26497249 PMC4718885

[13] Reding KM Schmidt PJ Rubinow DR Perimenopausal depression and early menopause: cause or consequence? Menopause (2017) 24:1333–5. 10.1097/GME.0000000000001016 29040218

[14] Schmidt P. J. Ben Dor R Martinez PE Guerrieri GM Harsh VL Thompson K. Effects of Estradiol Withdrawal on Mood in Women With Past Perimenopausal Depression: A Randomized Clinical Trial. JAMA Psychiatry (2015) 72:714–26. 10.1001/jamapsychiatry.2015.0111 26018333 PMC6391160

[15] Perich T Ussher J. Meade T. Menopause and illness course in bipolar disorder: A systematic review. Bipolar Disord. (2017) 19:434–43. 10.1111/bdi.12530 28796389

[16] Gordon JL Rubinow DR Eisenlohr-Moul TA Xia K Schmidt PJ Girdler SS Efficacy of transdermal estradiol and micronized progesterone in the prevention of depressive symptoms in the menopause transition: a randomized clinical trial. JAMA Psychiatry (2018) 5:149–57. 10.1001/jamapsychiatry.2017.3998 PMC5838629 29322164

[17] Zahn R Lythe KE Gethin JA Green S Deakin JF Workman C. Negative emotions towards others are diminished in remitted major depression. Eur Psychiatry (2015) 30:448–53. 10.1016/j.eurpsy.2015.02.005 25752724

[18] Stone EA Quartermain D Lin Y Lehmann ML. Central alpha1-adrenergic system in behavioral activity and depression. Biochem Pharmacol. (2007) 73:1063–75. 10.1016/j.bcp.2006.10.001 17097068

[19] Soares CN. Menopause and depression: keep your eye on the long run. Menopause (2016) 23:1272–4. 10.1097/GME.0000000000000791 27801703

[20] Xue W Wang W Gong T Zhang H Tao W Xue L PKA-CREB-BDNF signaling regulated long lasting antidepressant activities of Yueju but not ketamine. Sci Rep. (2016) 6:26331 10.1038/srep26331 27197752 PMC4873804

[21] Simeng G Wendong D Fushun W Effects of maternal deprivation at different lactation period on depression behavior and brain catecholamine of rats offsprings. Chin J Behav Med Brain Sci. (2014) 23:394–7.

[22] Wang P Li H Barde S Zhang MD Sun J Wang T. Depression-like behavior in rat: Involvement of galanin receptor subtype 1 in the ventral periaqueductal gray. Proc Natl Acad Sci U S A. (2016) 113:E4726–35. 10.1073/pnas.1609198113 27457954 PMC4987783

[23] Yang Y Cui Y Sang K Dong Y Ni Z Ma S. Ketamine blocks bursting in the lateral habenula to rapidly relieve depression. Nature (2018) 554:317–22. 10.1038/nature25509 29446381

[24] Cui Y Yang Y Ni Z Dong Y Cai G Foncell A. Astroglial Kir4.1 in the lateral habenula drives neuronal bursts in depression. Nature (2018) 554:323–7. 10.1038/nature25752 29446379

[25] Goel N Innala L Viau V. Sex differences in serotonin (5-HT) 1A receptor regulation of HPA axis and dorsal raphe responses to acute restraint. Psychoneuroendocrinology (2014) 40:232–41. 10.1016/j.psyneuen.2013.11.020 24485495

[26] Fox ME Studebaker RI Swofford NJ Wightman RM. Stress and drug dependence differentially modulate norepinephrine signaling in animals with varied HPA axis function. Neuropsychopharmacology (2015) 40:1752–61. 10.1038/npp.2015.23 25601230 PMC4915259

[27] Chhibber A Woody SK Karim Rumi MA Soares MJ Zhao L. Estrogen receptor beta deficiency impairs BDNF-5-HT2A signaling in the hippocampus of female brain: a possible mechanism for menopausal depression. Psychoneuroendocrinology (2017) 82:107–16. 10.1016/j.psyneuen.2017.05.016 28544903 PMC5523821

[28] Whedon JM KizhakkeVeettil A Rugo NA Kieffer KA. Bioidentical estrogen for menopausal depressive symptoms: a systematic review and meta-analysis. J Womens Health (2017) 26:18–28. 10.1089/jwh.2015.5628 27603786

[29] Kornstein SG Young EA Harvey AT Wisniewski SR Barkin JL Thase ME. The influence of menopause status and postmenopausal use of hormone therapy on presentation of major depression in women. Menopause (2010) 17:828–39. 10.1097/gme.0b013e3181d770a8 20616669 PMC2949279

[30] Walter EE Fernandez F Snelling M Barkus E. Stress induced cortisol release and schizotypy. Psychoneuroendocrinology (2018) 89:209–15. 10.1016/j.psyneuen.2018.01.012 29414034

[31] Roelfsema F Pijl H Keenan DM Veldhuis JD. Diminished adrenal sensitivity and ACTH efficacy in obese premenopausal women. Eur J Endocrinol. (2012) 167:633–42. 10.1530/EJE-12-0592 22909443

[32] Kok P Kok SW Buijs MM Westenberg JJ Roelfsema F Frölich M. Enhanced circadian ACTH release in obese premenopausal women: reversal by short-term acipimox treatment. Am J Physiol Endocrinol Metab. (2004) 287:E848–56. 10.1152/ajpendo.00254.2004 15280154

[33] Epperson CN Kim DR Bale TL. Estradiol modulation of monoamine metabolism: one possible mechanism underlying sex differences in risk for depression and dementia. JAMA Psychiatry (2014) 71:869–70. 10.1001/jamapsychiatry.2014.729 24898065 PMC4126841

[34] Shanmugan S Epperson CN. Estrogen and the prefrontal cortex: towards a new understanding of estrogen's effects on executive functions in the menopause transition. Hum Brain Mapp. (2014) 35:847–65. 10.1002/hbm.22218 23238908 PMC4104582

[35] Blair JA Bhatta S McGee H Casadesus G. Luteinizing hormone: Evidence for direct action in the CNS. Horm Behav. (2015) 76:57–62. 10.1016/j.yhbeh.2015.06.020 26172857 PMC4741372

[36] Wang L Zhou C Zhu D Wang X Fang L Zhong J. Serotonin-1A receptor alterations in depression: a meta-analysis of molecular imaging studies. BMC Psychiatry (2016) 16:319. 10.1186/s12888-016-1025-0 27623971 PMC5022168

[37] Taylor AE Munafo MR. Triangulating meta-analyses: the example of the serotonin transporter gene, stressful life events and major depression. BMC Psychol. (2016) 4:23. 10.1186/s40359-016-0129-0 27240561 PMC4886450

[38] Pascucci T Ventura R Latagliata EC Cabib S Puglisi-Allegra S. The medial prefrontal cortex determines the accumbens dopamine response to stress through the opposing influences of norepinephrine and dopamine. Cereb Cortex (2007) 17:2796–804. 10.1093/cercor/bhm008 17322559

[39] Howe MW Tierney PL Sandberg SG Phillips PE Graybiel AM. Prolonged dopamine signalling in striatum signals proximity and value of distant rewards. Nature (2013) 500:575–9. 10.1038/nature12475 23913271 PMC3927840

[40] Tye SJ Miller AD Blaha CD. Ventral tegmental ionotropic glutamate receptor stimulation of nucleus accumbens tonic dopamine efflux blunts hindbrain-evoked phasic neurotransmission: implications for dopamine dysregulation disorders. Neuroscience (2013) 252:337–45. 10.1016/j.neuroscience.2013.08.010 23962648

[41] Tye KM Mirzabekov JJ Warden MR Ferenczi EA Tsai H-C Finkelstein J. Dopamine neurons modulate neural encoding and expression of depression-related behaviour. Nature (2013) 493:537–41. 10.1038/nature11740 23235822 PMC4160519

[42] Baudry A Mouillet-Richard S Schneider B Launay JM Kellermann O. miR-16 targets the serotonin transporter: a new facet for adaptive responses to antidepressants. Science (2010) 329:1537–41. 10.1126/science.1193692 20847275

[43] Nieh EH Vander Weele CM Matthews GA Presbrey KN Wichmann R Leppla CA. Inhibitory input from the lateral hypothalamus to the ventral tegmental area disinhibits dopamine neurons and promotes behavioral activation. Neuron (2016) 90:1286–98. 10.1016/j.neuron.2016.04.035 27238864 PMC4961212

[44] Sharpe MJ Marchant NJ Whitaker LR Richie CT Zhang YJ Campbell EJ. Lateral hypothalamic GABAergic neurons encode reward predictions that are relayed to the ventral tegmental area to regulate learning. Curr Biol. (2017) 27:2089–100 e2085. 10.1016/j.cub.2017.06.024 28690111 PMC5564224

[45] Morales M Margolis EB. Ventral tegmental area: cellular heterogeneity, connectivity and behaviour. Nat Rev Neurosci. (2017) 18:73–85. 10.1038/nrn.2016.165 28053327

[46] Chauhan NR Kapoor M Prabha Singh L Gupta RK Chand Meena R Tulsawani R. Heat stress-induced neuroinflammation and aberration in monoamine levels in hypothalamus are associated with temperature dysregulation. Neuroscience (2017) 358:79–92. 10.1016/j.neuroscience.2017.06.023 28663093

